# The use of pulse pressure variation for predicting impairment of microcirculatory blood flow

**DOI:** 10.1038/s41598-021-88458-3

**Published:** 2021-04-28

**Authors:** Christoph R. Behem, Michael F. Graessler, Till Friedheim, Rahel Kluttig, Hans O. Pinnschmidt, Anna Duprée, E. Sebastian Debus, Daniel A. Reuter, Sabine H. Wipper, Constantin J. C. Trepte

**Affiliations:** 1 Department of Anesthesiology, Center of Anesthesiology and Intensive Care Medicine, University Medical Center Hamburg-Eppendorf, Martinistraße 52, 20246 Hamburg, Germany; 2 Department of Medical Biometry and Epidemiology, University Medical Center Hamburg-Eppendorf, Hamburg, Germany; 3 Department of Visceral- and Thoracic Surgery, Center of Operative Medicine, University Medical Center Hamburg-Eppendorf, Hamburg, Germany; 4 Department of Vascular Medicine, University Heart and Vascular Center Hamburg GmbH (UHZ), Hamburg, Germany; 5 Department of Anesthesiology and Intensive Care Medicine, Rostock University Medical Center, Rostock, Germany; 6 University Department for Vascular Surgery, Department of Operative Medicine, Medical University of Innsbruck, Innsbruck, Austria

**Keywords:** Translational research, Aneurysm, Aortic diseases

## Abstract

Dynamic parameters of preload have been widely recommended to guide fluid therapy based on the principle of fluid responsiveness and with regard to cardiac output. An equally important aspect is however to also avoid volume-overload. This accounts particularly when capillary leakage is present and volume-overload will promote impairment of microcirculatory blood flow. The aim of this study was to evaluate, whether an impairment of intestinal microcirculation caused by volume-load potentially can be predicted using pulse pressure variation in an experimental model of ischemia/reperfusion injury. The study was designed as a prospective explorative large animal pilot study. The study was performed in 8 anesthetized domestic pigs (German landrace). Ischemia/reperfusion was induced during aortic surgery. 6 h after ischemia/reperfusion-injury measurements were performed during 4 consecutive volume-loading-steps, each consisting of 6 ml kg^−1^ bodyweight^−1^. Mean microcirculatory blood flow (mean Flux) of the ileum was measured using direct laser-speckle-contrast-imaging. Receiver operating characteristic analysis was performed to determine the ability of pulse pressure variation to predict a decrease in microcirculation. A reduction of ≥ 10% mean Flux was considered a relevant decrease. After ischemia–reperfusion, volume-loading-steps led to a significant increase of cardiac output as well as mean arterial pressure, while pulse pressure variation and mean Flux were significantly reduced (Pairwise comparison ischemia/reperfusion-injury vs. volume loading step no. 4): cardiac output (l min^−1^) 1.68 (1.02–2.35) versus 2.84 (2.15–3.53), *p* = 0.002, mean arterial pressure (mmHg) 29.89 (21.65–38.12) versus 52.34 (43.55–61.14), *p* < 0.001, pulse pressure variation (%) 24.84 (17.45–32.22) versus 9.59 (1.68–17.49), *p* = 0.004, mean Flux (p.u.) 414.95 (295.18–534.72) versus 327.21 (206.95–447.48), *p* = 0.006. Receiver operating characteristic analysis revealed an area under the curve of 0.88 (CI 95% 0.73–1.00; *p* value < 0.001) for pulse pressure variation for predicting a decrease of microcirculatory blood flow. The results of our study show that pulse pressure variation does have the potential to predict decreases of intestinal microcirculatory blood flow due to volume-load after ischemia/reperfusion-injury. This should encourage further translational research and might help to prevent microcirculatory impairment due to excessive fluid resuscitation and to guide fluid therapy in the future.

## Introduction

Dynamic parameters of preload like pulse pressure variation (PPV) were shown to be capable of predicting macrohemodynamic responses to fluid administration^1,2^. This led to the recommendation to use these parameters for guiding fluid therapy in critically-ill patients both perioperatively as well as in intensive care unit settings^3–5^. The rationale for optimizing macrohemodynamics using PPV aims to increase cardiac output, assuming that this would result in improved tissue perfusion. However, coherence between macro- and microcirculation is frequently lost emphasizing the need for direct microcirculatory evaluation^6–8^.

An important aspect in guiding fluid therapy is that both hypovolemia but also particularly volume-overload have to be avoided^9,10^. In an earlier study in an experimental model of systemic inflammation we were able to demonstrate that already full utilization of preload reserve will result in volume-overload accompanied by impairments of microcirculatory blood flow and endothelial function^11^. This is clinically important since the occurrence of microcirculatory disturbances is a major cause for the development of multiple-organ failure and accounts for increased mortality^12–14^. Tissue perfusion and thus oxygenation depend on the perfusion of the microvasculature^6^. The effects of fluid administration on microcirculatory blood flow so far have not been sufficiently addressed. While some investigators have revealed promising results, other studies have failed to show an improvement of microcirculation due to fluid therapy, even if therapy was guided in accordance with macrohemodynamic goals^15–17^.

With regard to PPV, certain values can discriminate fluid-responders from non-responders, while also a zone of intermediate values with limited discriminatory power has been identified^18,19^. Beneath this zone an increase of stroke volume is rather unlikely, which on the other hand makes it all the more likely that fluid administration will promote volume-overload and consequently also a decrease of microcirculatory blood flow. This might be of particular interest in conditions of disturbed endothelial function and increased capillary leakage.

Therefore, we hypothesized that the underlying physiological principles of PPV could also be used to identify whether fluid administration results in a decrease of microcirculatory blood flow. We used a porcine model of ischemia/reperfusion injury during experimental aortic surgery as a model for a condition with microcirculatory disturbances, endothelial dysfunction and increased capillary leakage^20,21^. The aim of this study was to evaluate whether PPV does have the potential to predict an impairment of intestinal microcirculatory blood flow due to volume-load in our experimental setting.

## Results

### Study population

**Figure 1:**
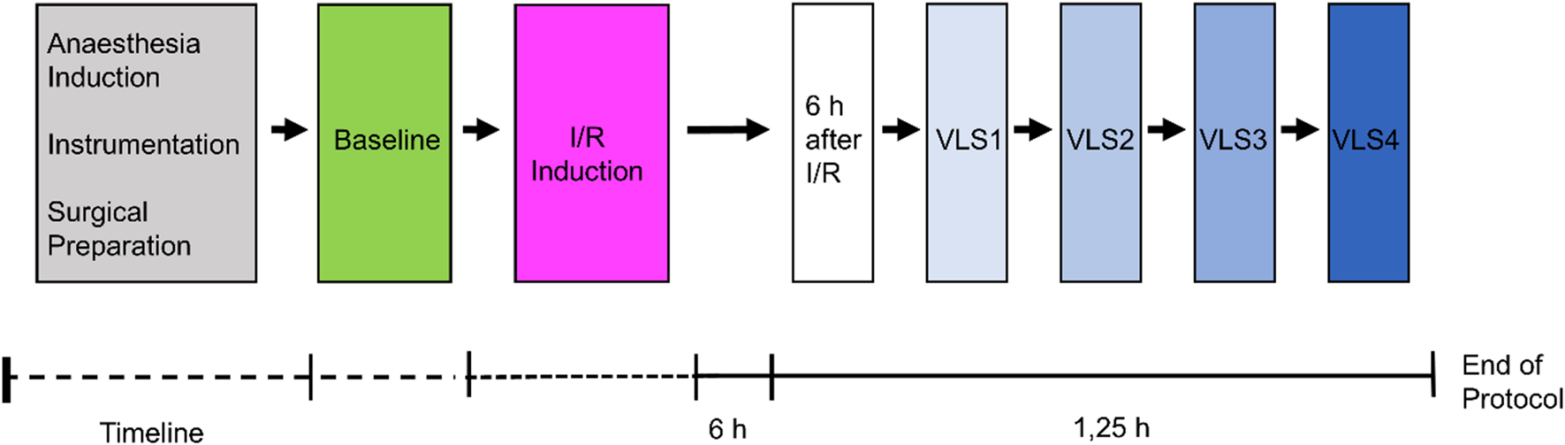
Experimental protocol. After completion of anesthesia induction, instrumentation and surgical preparations, baseline measurements were performed. Thereafter, ischemia/reperfusion (I/R) was induced. 6 h after ischemia/reperfusion induction, measurements were repeated and 4 consecutive volume-loading-steps (VLS) performed.

8 animals were studied. All animals survived until completion of protocol. No adverse events (e.g. severe bleeding, cardiac arrest) occurred. Mean body weight was 78.1 kg (95% CI 75.9–80.3). The study protocol is given in Fig. 1.

### Hemodynamic parameters

**Figure 2:**
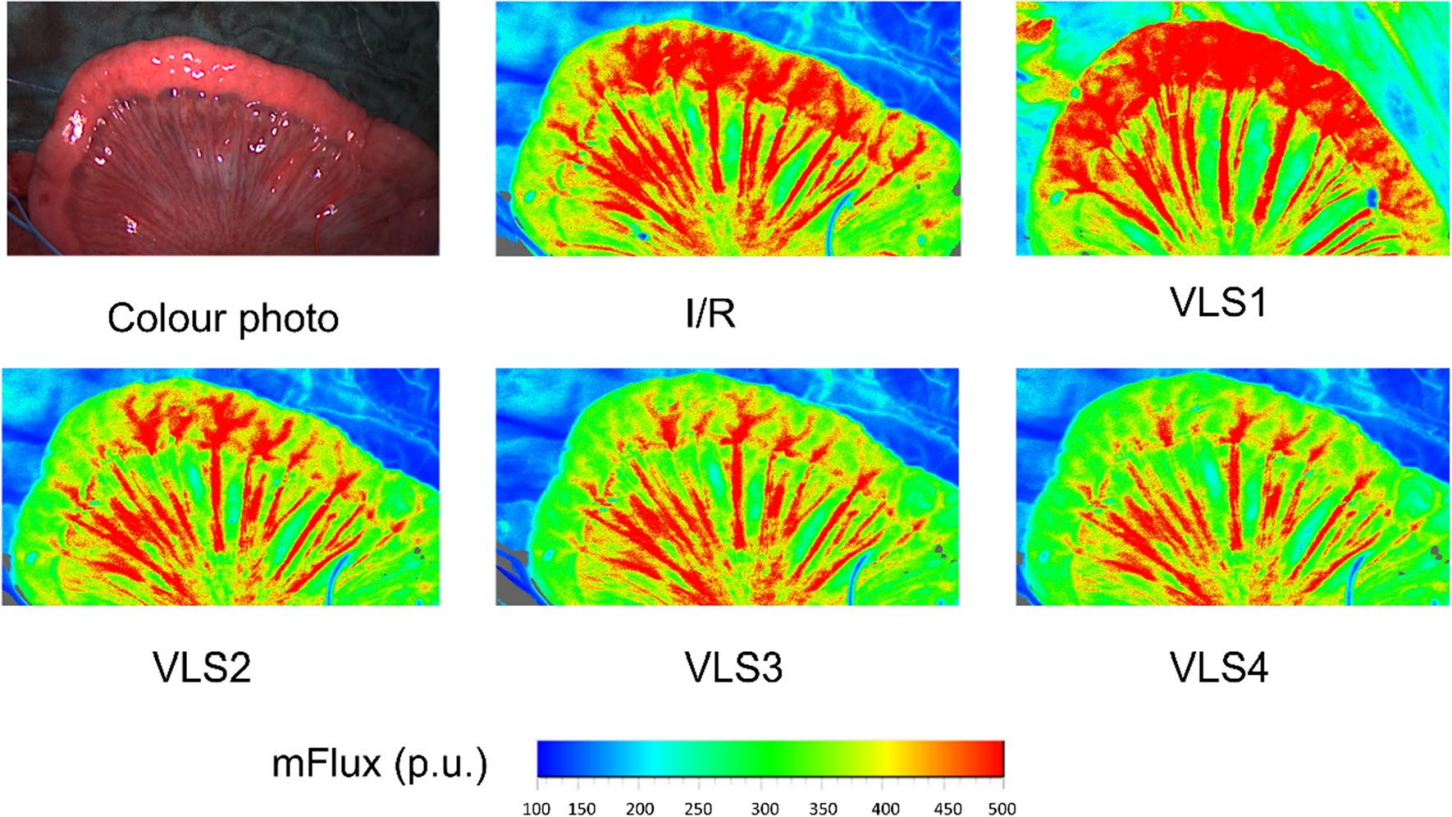
Sample picture of small intestine and color-coded examples of Laser-Speckle-Contrast-Imaging. Sample picture of exposed small intestine (ileum) and color-coded examples of Laser-Speckle-Contrast-Imaging derived mean intestinal microcirculatory blood flow (mFlux) at all points of measurement in an exemplary animal. I/R = ischemia/reperfusion; VLS = volume-loading-step.

**Figure 3:**
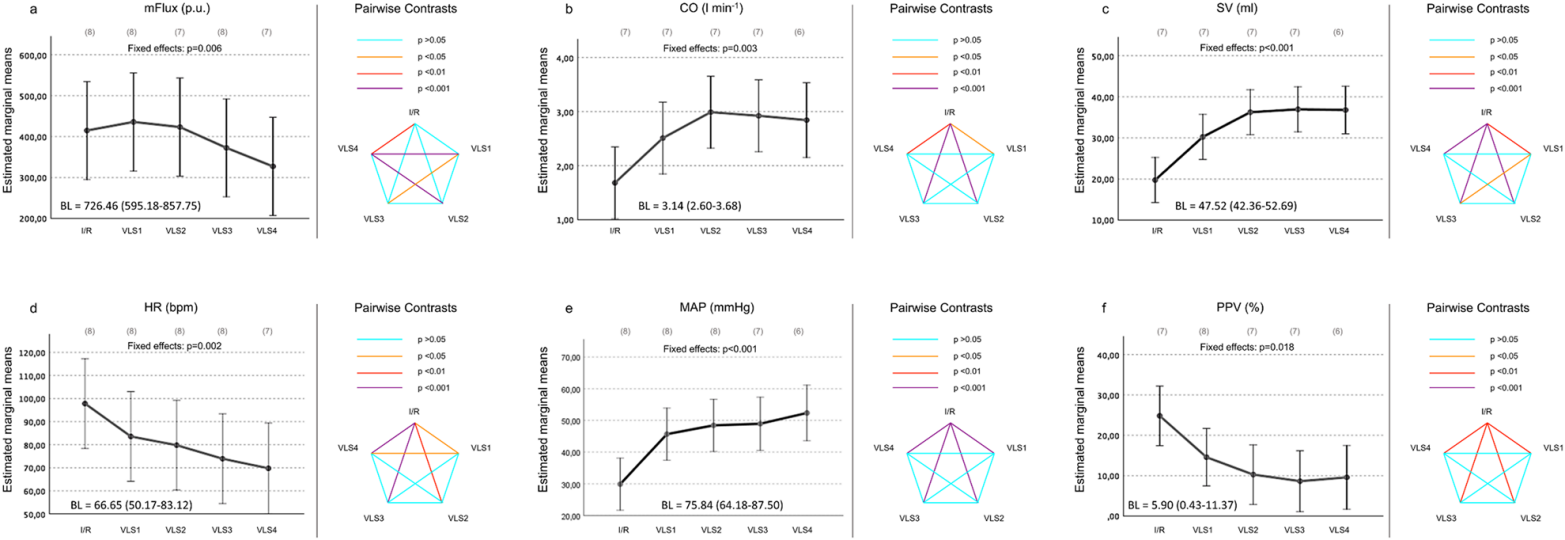
Changes of hemodynamic parameters throughout the experimental protocol. Changes throughout the experimental protocol of (**a**) mean microcirculatory blood flow (mFlux), (**b**) cardiac output (CO), (**c**) stroke volume (SV), (**d**) heart rate (HR), (**e**) mean arterial pressure (MAP) and (**f**) central pulse-pressure-variation (PPV). Data are presented as baseline adjusted estimated marginal means with 95% confidence intervals (left side). Results of pairwise comparisons are illustrated with color-coding of significance (right side). Points of measurements are 6 h after ischemia/reperfusion (I/R), volume loading steps 1–4 6 h after ischemia/reperfusion (VLS1-4). Number of valid values for each point of measurement are given in brackets. Baseline values (BL) prior ischemia/reperfusion are given in addition.

Sample pictures of intestinal mean Flux are given in Fig. 2. Baseline values *prior* ischemia/reperfusion as well as changes of micro- and macrohemodynamic parameters throughout the experimental protocol *after* ischemia/reperfusion are shown in Fig. 3.

### Prediction of intestinal microcirculatory blood flow decrease in ischemia/reperfusion

**Figure 4:**
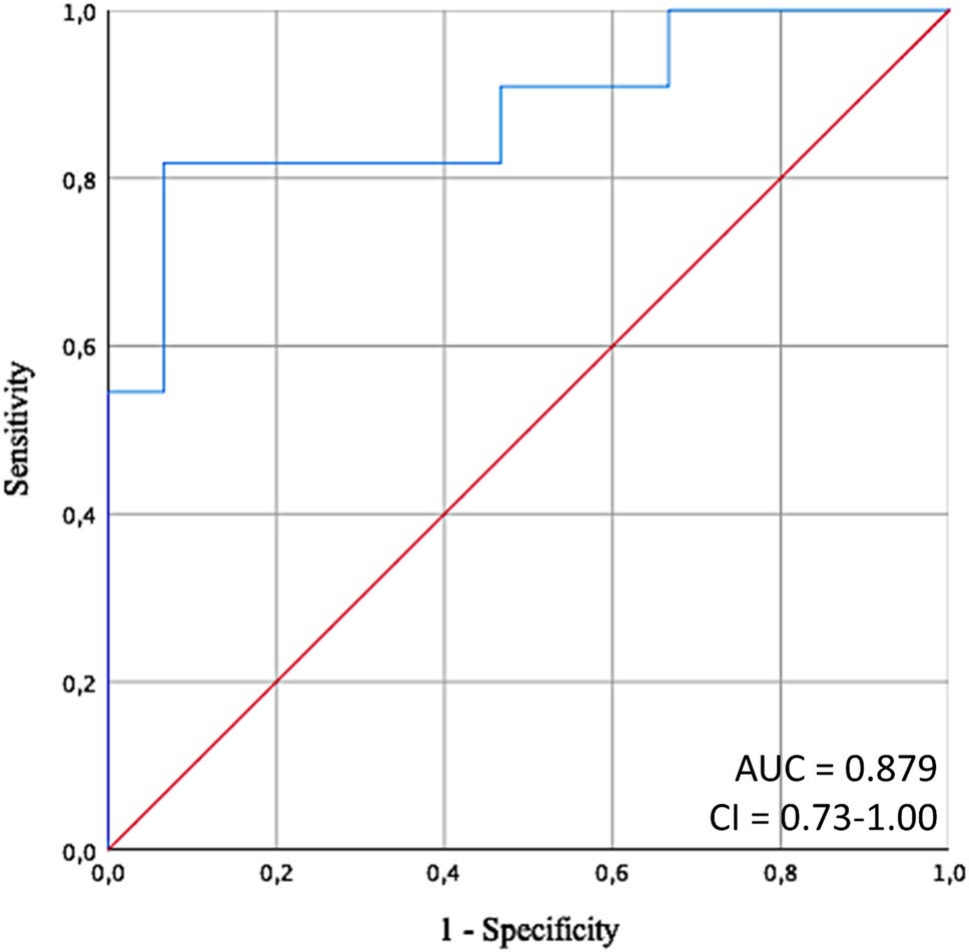
Receiver operating characteristic curve. Receiver operating characteristic curve to determine the ability of central pulse pressure variation to predict a ≥ 10% decrease of small intestinal microcirculation after ischemia/reperfusion. Direction of the test was set to decreasing values of pulse pressure variation. AUC = area under the curve, CI = 95% confidence interval.

In each animal 4 volume-loading-steps were performed resulting in a total of 32 volume-loading-steps. 3 volume-loading-steps had to be excluded due to artefacts in microcirculatory measurements. Of the remaining 29 volume-loading-steps, 3 PPV values had to be excluded due to cardiac arrhythmias. Thus, in total 26 volume-loading-steps were analyzed. There were 11 positive and 15 negative states. In detail for predicting decrease of intestinal microcirculatory blood flow in ischemia/reperfusion central PPV presented with an AUC of 0.88 (95% CI 0.74–1.00; *p* < 0.001). The receiver operating characteristic curve is presented in Fig. 4.

According to the receiver operating characteristic analysis in this experimental model of ischemia/reperfusion in pigs, the following cutoff-values for the prediction of microcirculatory decreases can be proposed: PPV ≤ 7.7% (Youden Index 0.75; sensitivity 81.82%, specificity 93.33%, positive predictive value 0.90, negative predictive value 0.88).

In addition, receiver operating characteristics analysis was performed using peripheral PPV. Peripheral PPV presented with an AUC of 0.83 (95% CI 0.66 to 1.00; *p* = 0.005) for prediction of intestinal microcirculatory blood flow decline with a cutoff value ≤ 8.0 (Youden Index 0.59, sensitivity 72.73%, specificity 86.67%, positive predictive value 0.80, negative predictive value 0.81). Details on receiver operating characteristics analysis are given as additional table 2  h in the additional file.

## Discussion

Our study demonstrates that PPV potentially could be used to predict decreases of intestinal microcirculatory blood flow due to excessive volume-load in an experimental setting of ischemia/reperfusion. Full utilization of preload reserve as reflected in low values for PPV aggravated impairment of intestinal microcirculatory flow. This suggests that dynamic indicators of preload may also be suitable to prevent microcirculatory deterioration when guiding fluid therapy in the future.

Over-infusion can bring about aggravated tissue edema leading to a deterioration of microcirculatory blood flow, while microcirculatory deterioration will result in oxygen deficits, multiple-organ failure and is associated with increased mortality^12–14^. Existing studies focusing on the effects of fluid administration on microcirculation often have shown a failure to improve microcirculation during fluid therapy and a saturation of the volume effect has been already described^15,16,22^. Therefore, identifying potential parameters that would enable prediction of microcirculatory behavior is of highest interest. Dynamic preload parameters such as pulse pressure variation can be used to assess fluid responsiveness^3,23–26^. Moreover, they may be useful to prevent volume-overload^19,27,28^. However, studies considering microcirculatory effects are scarce.

Loss of hemodynamic coherence has been described for different scenarios including perioperative medicine^6,7,29–31^. Microcirculatory alterations have a huge impact on prognosis and outcome not only in sepsis and heart failure, but also in surgical patients^29,31,32^. Moreover, anesthesia itself affects microcirculation^31,33,34^. Ischemia/reperfusion occurs in several medical conditions and leads to microcirculatory deterioration, accounts for the development of multiple-organ failure and is associated with increased mortality^12,13,20,21,35,36^. Alterations in microcirculatory blood flow have been shown to correlate with clinical outcome after liver, kidney and pancreas transplanation^31,37–39^. Ischemia/reperfusion induced by aortic cross clamping has been shown to influence microcirculation^31^. Loss of hemodynamic coherence with impairments of microcirculation as well as endothelial dysfunction has been described and microcirculation correlated with outcome following aortic cross clamping and vascular surgery^8,40–42^. In contrast, protection of splanchnic microcirculation with use of colloids has been shown to reduce complications in abdominal aortic surgery^43^. Intestinal microcirculatory alterations have also been shown to be associated with anastomotic leakage^31^. The small intestine is particularly susceptible to ischemia/reperfusion-injury resulting in intestinal-barrier dysfunction, bacterial translocation, systemic inflammation and multiple-organ failure^21,44,45^. Microcirculatory deteriorations are important determinants for manifestation of intestinal ischemia/reperfusion injury and have major prognostic impact^46^. The results of this study has revealed a promising new field of application for PPV as being capable of predicting impairments of intestinal microcirculatory blood flow in ischemia/reperfusion. Since effects of fluids on microvascular perfusion are associated with outcome^32^, use of PPV for prevention of small intestinal microcirculatory deterioration during fluid administration does have the potential to improve outcome.

In our experimental setting of ischemia/reperfusion PPV presented with a good AUC for predicting decline of microcirculatory blood flow due to volume-load. The cut-off value ≤ 7.7% in our series would be interpreted as being in the gray zone or to indicate and correspond to non-responders with regard to macrocirculatory responses to a fluid challenge. Cannesson and colleagues identified PPV-values between 9 and 13% as being inconclusive while Biais and others also showed that PPV-values between 4 and 17% failed to accurately predict fluid responsiveness^18,19^. Biais and others have reported, that this “grey zone” is already associated with the potential risk of over-infusion^19^.

Since most of clinical studies use PPV derived by femoral or radial artery blood pressure measurements^18,47^, peripheral PPV was assessed in addition. Although the AUC for peripheral PPV was lower, which may be explained be our experimental model using an aortic graft potentially affecting PPV distal of the graft^48^, it still presented with an acceptable AUC. Therefore, our results suggest that either central or peripheral PPV can be used for prediction of microcirculatory decline, which would facilitate translation to a clinical scenario.

Our results suggest that utilization of preload reserve after ischemia/reperfusion should be performed with particular caution avoiding fluid administration if stroke volume increase is uncertain as indicated by PPV-values below or inside the grey zone. In this regard, a prospective randomized clinical trial demonstrated an improved outcome using a fluid sparing regimen and limiting fluid boluses to presence of hypotension, low cardiac output and high stroke volume variation^27^. Biais and colleagues already tried to take into account the patients’ clinical condition according to PaO_2_/FiO_2_ ratios when trying to define optimal threshold values for PPV aiming to minimize the risk of volume-overload^19^. Volume-overload has clearly been shown to be associated with worsened outcome^10,49,50^. However, in patients at increased risk for complications during major abdominal surgery, a restrictive fluid regimen was not associated with improved survival and was associated with a higher rate of acute kidney injury^51^. Nevertheless, a fluid regimen tailored to microcirculatory demands may improve outcome, even if it is guided by macrohemodynamic parameters. An association between elevated central venous pressure and microcirculatory deterioration has already been described for septic patients^52^. Moreover, goal-directed therapy based on macrohemodynamic parameters has been shown to improve microcirculation in experimental pancreatitis, major surgery as well as abdominal surgery with important implications for outcome, and even in aortic surgery, prevention of microcirculatory decline could recently been shown by use of a goal directed hemodynamic management^53–56^. While hemodynamic coherence is frequently lost in various conditions^6,31^, association between pulse pressure variation and intestinal microcirculation may still exist. A potential association between pulse pressure variation and microcirculation may also be suggested by previous studies. While a loss of hemodynamic coherence was found for septic patients reflected by a dissociation between sublingual microcirculation and cardiac index as well as mean arterial pressure, this was only found in the late phase of sepsis. In the early phase of sepsis there was no dissociation between macro- and microcirculatory parameters. Most interesting, this was accompanied by a significant reduction of pulse pressure variation, while in the late phase, no significant change of pulse pressure variation was seen^16^. Moreover, an improvement of microcirculatory parameters following passive leg raise as well as fluid administration has already been shown for preload dependent septic patients^15^. In our project, there was a dissociation between the systemic variables cardiac output, stroke volume and mean arterial pressure, which has previously been discussed^57^. Nevertheless, pulse pressure variation predicted microcirculatory decline. The results of our study are also in line with the results of a recent study conducted in patients undergoing major surgery by Bouattour et al.. In this clinical study, the authors could demonstrate that fluid administration in conditions of preload dependency as identified by a pulse pressure variation > 13% led to an improved microcirculation as assessed with sublingual sidestream darkfield imaging^47^. These results underline the usefulness of PPV to guide treatment of microcirculation. Based on the ability of PPV to predict microcirculatory decline due to fluid administration as shown in this study, we propose that in ischemia/reperfusion conditions, a PPV-based fluid management may help to protect intestinal microcirculation.

Our study has certain limitations. First, we limited the number of animals in this hypothesis generating pilot study. Sensible a priori input data to be used for sample size and power calculations were not available, hence, we chose a sample size that appeared feasible with respect to size of the study protocol and in accordance to ARRIVE and FELASA guidelines as well as 3R-principles reducing animal numbers to a reasonable sample size while being able to address the scientific topic properly^58^. Moreover, the number of animals as well as volume-loading-steps is comparable with existing studies^59–64^. In addition, the number of volume-loading-steps is also comparable to a number of clinical studies^65–72^. As this was an pilot study for generating hypotheses rather than confirmatory test pre-specified hypotheses, alpha error adjustment for multiple testing was not done. In addition, we did not include a control group. However, we assumed that the risk for microcirculatory deterioration due to fluid therapy would be low in healthy conditions and that there would be to few positive states to reliably assess the ability of PPV for prediction of microcirculatory decline in a control group. Another limitation would be that duration of vessel ischemia was not intended as standardized ischemia times but was dependent on the surgical techniques used. Although ischemic times were different between animals, the variation of vessel ischemia would reflect the clinical situation of open thoraco-abdominal aortic repair, especially since surgical procedures were performed by experienced vascular surgeons and the advantage of the study would therefore be the comparability to the clinical scenario^73–75^. Moreover, besides predicting decreases of intestinal microcirculatory blood flow caused by volume-overload, the clinical impact of this microcirculatory decline was not further assessed in our study. However, this has already been shown by previous publications^31,32^. Choice of anesthesia can also influence microcirculation^31^. Therefore, we have used a standardized approach with identical anesthesia doses and identical ventilation settings. Moreover, in a recent study on the effects of total intravenous vs. balanced anesthesia, use of a goal-directed therapy prevented deterioration of microcirculatory perfusion as well as oxygenation for both groups^56^. Therefore, our results may also be translated to patients receiving balanced anesthesia. Different effects of vasoactive drugs on microcirculation have been described^31,76^. Although all vasopressors used during surgical phases have been terminated for at least 30 min prior measurements, we cannot rule out residual effects of vasopressors even if they are very much unlikely after 30 min of cessation. While the choice of fluids does not influence the predictive value of PPV^26^, colloids may be different from crystalloids in regard to microcirculation. While some authors found no benefits or even detrimental effects for colloids, others have found beneficial effects in regard to microcirculation^27,31,77–89^. Choice of fluids was based on the experience from previous studies showing pronounced effects of hydroxyethyl-starch colloids on macro- as well as microcirculation in our porcine models^11,53,90–94^. Moreover, detrimental effects on microcirculation have been described for crystalloids as well^95–98^. Nevertheless, future studies are needed to test the ablity of pulse pressure varation to predict microcirculatory decline during fluid administration with crystalloids as well. In regard to laser speckle contrast imaging it should be noted that capillaries have a diameter of about 7 µm^99,100^, while laser speckle contrast imaging technique has a maximum resolution of about 10 µm, so it is barely possible to directly visualize capillaries with laser speckle contrast imaging. However, it is possible to detect perfusion in a capillary bed even when capillaries cannot be identified individually^100^. Nevertheless, the limitation of the used method for microcirculatory evaluation should be regarded. Future studies should also investigate the usefulness of dynamic preload parameters for maintenance and optimization of microcirculation in different clinical settings and other organs.

In conclusion, the results of our study show that PPV does have the potential to predict decreases of intestinal microcirculatory blood flow caused by volume-load in an experimental setting of ischemia/reperfusion. Full utilization of preload as reflected in low values of PPV resulted in impaired microcirculatory blood flow. This should encourage further translational research and might help to prevent microcirculatory impairment due to excessive fluid resuscitation and to guide fluid therapy in the future.

## Methods

### Study design

The study was conducted as a prospective explorative pilot study in 8 anesthetized domestic pigs (German landrace) both female and male using animals weighing approximately 75–80 kg. This study was performed in combination with a feasibility study for a new hybrid-graft implantation in accordance to ARRIVE and FELASA guidelines and 3Rs-principles reducing animal number. The animals received care in compliance with the ‘Guide for the Care and Use of Laboratory Animals’ (NIH publication No. 86–23, revised 2011) as well as FELASA guidelines and recommendations and experiments were carried out according to the ARRIVE guidelines^58,101^. Please see the ARRIVE guidelines checklist as well as additional comments on the ARRIVE guidelines given in the additional file. Results on microcirculatory behavior have been previously reported and methods have been previously described^57,102^.

The study protocol is given in Fig. 1. This study was performed in coherence with current recommendations on the use of pulse pressure variation: All animals were mechanically ventilated with tidal volumes of 8 ml kg^−1^ total body weight, arrhythmic episodes were excluded and high respiratory rates avoided^103,104^. Ischemia/reperfusion was induced during aortic hybrid-graft implantation. Micro- and macrocirculation were measured at baseline *prior* ischemia/reperfusion and 6 h *after* ischemia/reperfusion induced by aortic hybrid-graft implantation. Thereafter 4 consecutive volume-loading-steps were performed followed by micro- and macrocirculatory measurements. Each volume-loading-step consisted of 6 ml kg^−1^ bodyweight^−1^ colloids (Voluven 6%, Fresenius Kabi, Bad Homburg, Germany). Each volume-loading-step was performed during a time period of 5 min using pressurized infusions. After completion of each volume-loading-step, 5 min were allowed for equilibration. At least 30 min prior measurements, all vasopressors used during the surgical phase were terminated to exclude effects of these substances.

### Assessment of microcirculation

Microcirculatory blood flow was directly assessed using laser speckle contrast imaging as previously described^57^. Laser speckle contrast imaging has been frequently used for monitoring of the microcirculation including intestinal microcirculation as well models of ischemia/reperfusion^105–115^. For use of laser speckle contrast imaging, tissues are illuminated with coherent laser light. The backscattered light from the tissue then forms a random interference pattern at the detector. This interference pattern is called speckle pattern^106^. Laser speckle contrast imaging is based on the laser Doppler method, however, it has a much higher spatial and temporal resolution with use of full field laser measurements. It allows imaging of large surface area, is contact-free and can measure perfusion in real time^111^. It can detect mean microcirculatory blood flow (mFlux) up to a tissue depth of 3 mm. It is non-invasive and has good reproducibility^115^. The mean Flux (mFlux) is a dimensionless unit of microcirculatory blood flow, with the unit p.u.^112^. In detail, the speckle-laser (MoorFLPI-2, Moor Instruments, Axminster, UK) was positioned 25 cm above the intestinal segment using a target laser. For each measurement step microcirculatory blood flow was assessed for a 30 s period reducing variability due to respiration and organ movement^57,111,116^. A blinded investigator defined the region of interest off-line and calculated the mean Flux for the region of interest using a dedicated software (Moor FLPI-2 Review Software, v. 4.0, Moor Instruments, Axminster, UK)^57^.

### Assessment of macrocirculation

Cardiac output and stroke volume were assessed invasively using a perivascular flow probe (Confidence PAU Flowprobe, chronic liner, 16 or 18 mm, Transonic Systems Inc., Ithaca, NY, USA) that was fit around the descending aorta and connected to the adapted hardware (Perivascular Flow Module, Transonic Systems Inc., Ithaca, NY, USA). Invasive pressure catheters (Millar Micro-Tip pressure catheters, Houston, Texas, USA) for assessment of arterial pressure were directly inserted into the ascending aorta via the carotid artery for arterial pressure measurement and assessment of central PPV as well as in the femoral artery for assessment of peripheral PPV using 8 Fr. introducer sheaths. Macrohemodynamic measurements were recorded simultaneously with microcirculatory assessment for a period of 2 min.

### Data acquisition and processing

Invasive data were recorded by adapted hardware from ADInstruments (ADInstruments Bridge Amp and PowerLab, ADInstruments Ltd., Oxford, UK) and Transonic (Perivascular Flow Module, Transonic Systems Inc., Ithaca, NY, USA). Data analysis was performed off-line using LabChart software (LabChart Pro, version 8, ADInstruments Ltd., Oxford, UK). Central and peripheral PPV were calculated using data from 10 respiratory cycles. The following formula was applied: ${\text{PULSE PRESSURE VARIATION }}\left( {\text{\% }} \right) = \frac{{\left( {Maximum Pulse Pressure - Minimum Pulse Pressure} \right)}}{{\left( {Mean Pulse Pressure} \right)}}*100$

### Animal care, anesthesia and surgical procedures

Animal care, anesthesia and surgical procedures have been previously described^57,102^. Animals were brought to animal care facilities at least 7 days prior experiments and were housed in accordance to animal welfare recommendations. Animals were given food and water ad libitum and health status was regularly assessed by the responsible veterinarian. A fasting time of 12 h was maintained prior to the experiments^102^. Anesthesia was performed intravenously using a combination of an opiod together with different hypnotic agents to secure deep anesthesia during the entire protocol as previously described^57^. For anesthesia induction all animals received intramuscular injections of ketamine 10 mg kg^−1^ bodyweight^−1^, azaperone 4 mg kg^−1^ bodyweight^−1^, atropine 0.01 mg kg^−1^ bodyweight^−1^ and midazolam 10 mg for premedication. Thereafter, all animals were surgically tracheotomized and the trachea was intubated via the tracheotomy ostium. Maintenance of anesthesia was performed by continuous infusion of propofol (4 mg kg^−1^ bodyweight^−1^ h^−1^), fentanyl (10 µg kg^−1^ bodyweight^−1^ h^−1^), midazolam (0.3 mg kg^−1^ bodyweight^−1^ h^−1^) and ketamine (6 mg kg^−1^ bodyweight^−1^ h^−1^). This combination provides adequate anesthesia as well as hemodynamic stability and has been successfully used in previous studies^57,117,118^. Adequacy of anesthesia was assessed by careful observation of vital signs and ventilation parameters as well as by absence of any movements during the entire protocol with special attendance to phases of surgical stimulus. Additional bolus doses of fentanyl (50 µg) were given if there was any indication of pain or distress^102^. Pancuronium (0.1 mg kg^−1^ bodyweight^−1^) was only given for tracheotomy. A volume-controlled ventilation was established using tidal volumes of 8 ml kg^−1^ bodyweight^−1^ and a positive end-expiratory pressure of 8 cmH_2_O and ventilator frequency was adjusted to maintain an end-expiratory carbon dioxide tension (etCO_2_) of 35–40 mmHg (Julian, Dräger Medical, Lübeck, Germany). All animals were placed in supine position on a warming blanket to prevent heat loss^57,102^. Surgical procedures have been previously described^57,118^ and are given in detail in the additional file. Duration of vessel ischemia was not intended as standardized ischemia times but was dependent on the surgical techniques used. Times of vessel ischemia are given as additional table 1 in the additional file. The entire experimental procedure and handling of the animals was supervised by the responsible veterinarian.

### Euthanasia

After completion of the study protocol all animals were sacrificed during deep anesthesia by fast injection of 40 mmol potassium chloride as previously described^57,102^.

### Sample size calculation

As previously described, a priori input data to be used for sample size and power calculations were not available, hence, a sample size was chosen that appeared feasible with respect to size of the study protocol and in accordance to ARRIVE and FELASA guidelines and 3R-principles reducing animal numbers to a reasonable sample size while being able to address the scientific topic properly^57^.

### Primary endpoint

Primary endpoint of this study was the ability of PPV to predict a mean microcirculatory flux decrease ≥ 10% following fluid administration.

### Statistical analysis

The statistical plan was approved by all authors before start of the study. The dependent variables cardiac output, stroke volume, heart rate, mean arterial pressure, central PPV and mean Flux were subjected to general linear mixed model analyses, using the SPSS v. 24 routine GENLINMIXED for continuous data with an identity link function as previously described^57^. Models were specified with fixed effects for variable measurement point and random intercepts for animals. In addition, baseline values prior ischemia/reperfusion were included as fixed effects to the model as well to adjust for differences in baseline values between animals. Measurement points were considered as repeated measures within animals. Marginal means with 95% CI were computed for all dependent variables at all measurement points, followed by multiple pairwise comparisons of measurement point means via least significant difference tests. The assessment of the ability of PPV to predict microcirculatory decline was performed by calculating receiver operating characteristic curves. The response to fluid administration was considered significant, if mean microcirculatory flux decreased by at least 10%. Direction of the test was set to decreasing values of PPV, assuming that the likelihood of the state event increases with decreases in PPV. The value of the predictor variable resulting in a maximum Youden index^119^ was assumed to represent its ideal cutoff value. Statistical analyses were performed using the SPSS statistical software package 24 (IBM SPSS Statistics Inc., USA). Additional details on statistical analysis are given in the in the additional file as additional tables 2a–h. Variables are expressed as mean (95% confidence interval, CI). Two-tailed *p* values less than 0.05 were considered significant. In addition, results of general linear mixed model analyses that handled baseline values as part of the outcome and not as fixed effect covariate are given as additional table 3, along with *p* values of pairwise contrasts.

### Ethics approval and consent to participate

The study was approved by the Governmental Commission on the Care and Use of Animals of the City of Hamburg (Reference-No. 101/15). The animals received care in compliance with the ‘Guide for the Care and Use of Laboratory Animals’ (NIH publication No. 86–23, revised 2011) as well as FELASA guidelines and recommendations and experiments were carried out according to the ARRIVE guidelines. Please see the ARRIVE guidelines checklist as well as additional comments on the ARRIVE guidelines given in the additional file.

## Supplementary Information

Supplementary Information

## Data Availability

The datasets analysed during the current study are available from the corresponding author on reasonable request. Additional details on statistical analysis are given in the additional file.

## Abbreviation

PPV: Pulse pressure variation
