# Supplementary material for: The use of pulse pressure variation for predicting impairment of microcirculatory blood flow
Source: Sci Rep. 2021 Apr 28;11:9215. doi: 10.1038/s41598-021-88458-3 (PMC8080713; doi:10.1038/s41598-021-88458-3)
Supplement: Supplementary file 1 — Supplementary Information [file 41598_2021_88458_MOESM1_ESM.pdf]

Additional files for manuscript:

The use of pulse pressure variation for predicting impairment of microcirculatory blood flow

Christoph R. Behem[a], M.D.; Michael F. Graessler[a], M.D.; Till Friedheim[a], M.D.; Rahel Kluttig[a]; M.D.; Hans O. Pinnschmidt[b], Ph.D.; Anna Duprée[c], M.D.; E. Sebastian Debus[d], M.D., Ph.D.;Daniel A. Reuter[e], M.D., Ph.D.; Sabine H. Wipper[f], M.D., Ph.D.; Constantin J. C. Trepte[a], M.D., Ph.D.

a) Department of Anesthesiology, Center of Anesthesiology and Intensive Care Medicine, University Medical Center Hamburg-Eppendorf, Hamburg, Germany
b) Department of Medical Biometry and Epidemiology, University Medical Center Hamburg-Eppendorf, Hamburg, Germany
c) Department of Visceral- and Thoracic Surgery, Center of Operative Medicine, University Medical Center Hamburg-Eppendorf, Hamburg, Germany
d) Department of Vascular Medicine, University Heart and Vascular Center Hamburg GmbH (UHZ), Hamburg, Germany
e) Department of Anesthesiology and Intensive Care Medicine, Rostock University Medical Center, Rostock, Germany
f) University Department for Vascular Surgery, Department of Operative Medicine, Medical University of Innsbruck, Austria

Additional file 1: ARRIVE Guidelines Checklist

Additional file 1: ARRIVE Guidelines Checklist

ARRIVE

The ARRIVE Guidelines Checklist

Animal Research: Reporting In Vivo Experiments

Carol Kilkenny[1], William J Browne[2], Innes C Cuthill[3], Michael Emerson[4] and Douglas G Altman[5]

[1]The National Centre for the Replacement, Refinement and Reduction of Animals in Research, London, UK, [2]School of Veterinary Science, University of Bristol, Bristol, UK, [3]School of Biological Sciences, University of Bristol, Bristol, UK, [4]National Heart and Lung Institute, Imperial College London, UK, [5]Centre for Statistics in Medicine, University of Oxford, Oxford, UK.

	ITEM	RECOMMENDATION	Section/ Paragraph
Title	1	Provide as accurate and concise a description of the content of the article as possible.	Title page (Page 1)
Abstract	2	Provide an accurate summary of the background, research objectives, including details of the species or strain of animal used, key methods, principal findings and conclusions of the study.	Abstract (Page 3-4)
INTRODUCTION			
Background	3	a. Include sufficient scientific background (including relevant references to previous work) to understand the motivation and context for the study, and explain the experimental approach and rationale.<br>b. Explain how and why the animal species and model being used can address the scientific objectives and, where appropriate, the study's relevance to human biology.	Introduction (Page 5-6)
Objectives	4	Clearly describe the primary and any secondary objectives of the study, or specific hypotheses being tested.	Introduction (Page 6)
METHODS			
Ethical statement	5	Indicate the nature of the ethical review permissions, relevant licences (e.g. Animal [Scientific Procedures] Act 1986), and national or institutional guidelines for the care and use of animals, that cover the research.	Methods (Page 16); Declarations (Page 39)
Study design	6	For each experiment, give brief details of the study design including:<br>a. The number of experimental and control groups.<br>b. Any steps taken to minimise the effects of subjective bias when allocating animals to treatment (e.g. randomisation procedure) and when assessing results (e.g. if done, describe who was blinded and when).<br>c. The experimental unit (e.g. a single animal, group or cage of animals).<br>A time-line diagram or flow chart can be useful to illustrate how complex study designs were carried out.	Methods (Page 16); Figure 1
Experimental procedures	7	For each experiment and each experimental group, including controls, provide precise details of all procedures carried out. For example:<br>a. How (e.g. drug formulation and dose, site and route of administration, anaesthesia and analgesia used [including monitoring], surgical procedure, method of euthanasia). Provide details of any specialist equipment used, including supplier(s).<br>b. When (e.g. time of day).<br>c. Where (e.g. home cage, laboratory, water maze).<br>d. Why (e.g. rationale for choice of specific anaesthetic, route of administration, drug dose used).	Methods (Page 16-20); Additional File
Experimental animals	8	a. Provide details of the animals used, including species, strain, sex, developmental stage (e.g. mean or median age plus age range) and weight (e.g. mean or median weight plus weight range).<br>b. Provide further relevant information such as the source of animals, international strain nomenclature, genetic modification status (e.g. knock-out or transgenic), genotype, health/immune status, drug or test naïve, previous procedures, etc.	Methods (Page 16); Results (Page 7)

The ARRIVE guidelines. Originally published in *PLoS Biology*, June 2010[1]

Housing and husbandry	9	Provide details of:<br>a. Housing (type of facility e.g. specific pathogen free [SPF]; type of cage or housing; bedding material; number of cage companions; tank shape and material etc. for fish).<br>b. Husbandry conditions (e.g. breeding programme, light/dark cycle, temperature, quality of water etc for fish, type of food, access to food and water, environmental enrichment).<br>c. Welfare-related assessments and interventions that were carried out prior to, during, or after the experiment.	Methods (Page 16-20)
Sample size	10	a. Specify the total number of animals used in each experiment, and the number of animals in each experimental group.<br>b. Explain how the number of animals was arrived at. Provide details of any sample size calculation used.<br>c. Indicate the number of independent replications of each experiment, if relevant.	Methods (Page 20)
Allocating animals to experimental groups	11	a. Give full details of how animals were allocated to experimental groups, including randomisation or matching if done.<br>b. Describe the order in which the animals in the different experimental groups were treated and assessed.	n.a.
Experimental outcomes	12	Clearly define the primary and secondary experimental outcomes assessed (e.g. cell death, molecular markers, behavioural changes).	Methods (Page 20)
Statistical methods	13	a. Provide details of the statistical methods used for each analysis.<br>b. Specify the unit of analysis for each dataset (e.g. single animal, group of animals, single neuron).<br>c. Describe any methods used to assess whether the data met the assumptions of the statistical approach.	Methods (Page 20-21) Additional file
RESULTS			
Baseline data	14	For each experimental group, report relevant characteristics and health status of animals (e.g. weight, microbiological status, and drug or test naïve) prior to treatment or testing. (This information can often be tabulated).	Results (Page 7)
Numbers analysed	15	a. Report the number of animals in each group included in each analysis. Report absolute numbers (e.g. 10/20, not 50%[2]).<br>b. If any animals or data were not included in the analysis, explain why.	Results (Page 7-8) Figures 3 & 4
Outcomes and estimation	16	Report the results for each analysis carried out, with a measure of precision (e.g. standard error or confidence interval).	Results (Page 7-8) Figures 3 & 4
Adverse events	17	a. Give details of all important adverse events in each experimental group.<br>b. Describe any modifications to the experimental protocols made to reduce adverse events.	Results (Page 7)
DISCUSSION			
Interpretation/ scientific implications	18	a. Interpret the results, taking into account the study objectives and hypotheses, current theory and other relevant studies in the literature.<br>b. Comment on the study limitations including any potential sources of bias, any limitations of the animal model, and the imprecision associated with the results[2].<br>c. Describe any implications of your experimental methods or findings for the replacement, refinement or reduction (the 3Rs) of the use of animals in research.	Discussion (Page 9-15)
Generalisability/ translation	19	Comment on whether, and how, the findings of this study are likely to translate to other species or systems, including any relevance to human biology.	Discussion (Page 9-15)
Funding	20	List all funding sources (including grant number) and the role of the funder(s) in the study.	Declarations (Page 40)

References:

1. Kilkenny C, Browne WJ, Cuthill IC, Emerson M, Altman DG (2010) Improving Bioscience Research Reporting: The ARRIVE Guidelines for Reporting Animal Research. *PLoS Biol* 8(6): e1000412. doi:10.1371/journal.pbio.1000412
2. Schulz KF, Altman DG, Moher D, the CONSORT Group (2010) CONSORT 2010 Statement: updated guidelines for reporting parallel group randomised trials. *BMJ* 340:c332.

Additional file 2: Additional comments on ARRIVE Guidelines

This document contains additional comments on ARRIVE Guidelines.

Additional comments on ARRIVE Guidelines:

1. The abstract includes a summary of background, objectives and gives specific information to the species and methods used as requested by the ARRIVE Guidelines.
2. In the introduction we have included an overview of the scientific background and previous work relevant to this subject to provide adequate information to explain the purpose of this study. We have included statements on the usefulness of our experimental model and clearly stated the aim of this study as requested by the ARRIVE Guidelines.
3. In the declaration we have included information on ethical approval including reference-no. and have referred to the ARRIVE Guidelines, FELASA guidelines and recommendations as well as NIH-guidelines on animal care in the methods. We have explained how we were able to reduce animal number according to 3Rs principles by combining different studies. Information on animal number, a detailed description of the used methods as well as measures to reduce bias are stated in the methods section as requested by the ARRIVE Guidelines. We further included a figure to illustrate the course of the study. A detailed description of anesthesia, hemodynamic management and euthanasia is given in the methods section. Details on surgical preparation is given in the Additional File 3. The method section includes details on the animals used including race, weight, and animal care prior experiments. Sample size justification is commented on in the statistic section as well as in the limitation section. We included a detailed prescription of the methods deriving outcome measures and the statistical analysis is described in adequate detail.
4. Details on adverse events and animal survival during experiments is given in the results, the exact absolute numbers for any values are given in the results section and figures and we have explained exclusion of measurements in detail. Results are reported with CI as a measure of precision.
5. In the discussion, we have interpreted the results in regard to study objectives and hypotheses and have compared them with relevant existing studies. We have clearly commented on potential sources of bias in the limitations section of the discussion, have commented on the translational value of this study and have included a statement on funding.

Additional file 3: Details of surgical procedures

This file contains details of surgical procedures.

Details of surgical procedures:

Surgical preparation was carried out according to standardized preparation techniques. The carotid artery was exposed and an 8 Fr. introducer sheath was inserted for placement of a Millar catheter in the ascending aorta. The femoral artery was exposed and an 8 Fr. introducer sheath was inserted for placement of a Millar catheter in the femoral artery. A 7.5 Fr. venous catheter and 8 Fr. introducer sheath were inserted in the right sided internal jugular vein for volume and drug application. A median laparotomy was performed. The descending aorta was exposed for graft implantation and a flow-probe was placed around the descending aorta proximal of the graft landing zone. The peritoneum was fenestrated in the area of the terminal ileum. A 15-20 cm segment of the terminal ileum was defined for measurement of intestinal microcirculatory blood flow. The chosen ileum segment was marked with loosely attached vessel loops for identification whereat meticulous caution was taken not to affect perfusion due to tension or compression of the segment. Between baseline measurement and the measurement 6 h after ischemia/reperfusion, the ileum segment was left intraperitoneal. For Laser-Speckle-Contrast-Imaging measurements the ileum segment was carefully led on a humidified compress outside the peritoneum. 10 minutes were allowed for stabilization after extraperitoneal placement of the segment. Meticulous caution was paid not to distort mesenteric vessels and before each measurement the segment was carefully checked for perfusion defects. Size of peritoneal fenestration was chosen wide enough not to impair perfusion and eventually enlarged if necessary. Between the volume loading steps after ischemia/reperfusion,

the ileum segment was covered with humidified and warmed compresses to prevent heat and moisture loss. Undisturbed perfusion and lack of any compression or tension was carefully checked prior each measurement.

Aortic hybrid-graft implantation was performed using a hybrid-graft device combining a proximal stent graft and a distal multi-branched graft for re-implantation of the coeliac trunk (TC), the superior mesenteric artery (SMA) and both renal arteries (LRA, RRA). The infra-diaphragmatic aorta was exposed through retroperitoneal access. After infra-coeliac cross-clamping, the TC was divided and the proximal stent-grafted part of the graft was introduced into the descending aorta via the former coeliac ostium and carefully extracted. The coeliac, superior mesenteric, and renal arteries were successively connected to the corresponding side branches of the graft. Finally, iliac arteries (right iliac artery (RIA) and left iliac artery (LIA)) were anastomosed. Times of ischemia for TC, SMA, RRA, LRA, RIA, LIA are given as Additional File (Additional Table 1).

Additional file 4: Additional Tables

This file contains information on vessel ischemia duration (Additional Table 1) as well as statistical details (Additional Table 2a-h). In addition, pairwise comparison of macro- and microcirculation with inclusion of baseline values are given in Additional Table 3.

Additional Table 1

Duration of vessel ischemia was not intended as standardized ischemia times but was dependent on the surgical techniques used. Additional Table 1 presents mean ischemic times for the different vessels.

Additional Table 1	TC	AMS	RRA	LRA	LIA	RIA
Mean Ischemic times (min.) (95% CI)	12.57 (11.36-13.78)	9.86 (8.38-11.34)	13.07 (10.48-15.67)	25.29 (18.56-32.01)	17.21 (11.73-22.70)	18.14 (4.33-31.96)

Additional Table 1: Ischemic times in minutes for different vessels. Data are presented as means with 95% confidence intervals. TC = coeliac trunk, AMS = superior mesenteric artery, RRA = right renal artery, LRA = left renal artery, LIA = left iliac artery, RIA = right iliac artery.

Additional Table 2a-h

Additional Table 2 a-h contain detailed information on general linear mixed model analyses for mean Flux (2a), cardiac output (2b), stroke volume (2c), heart rate (2d), mean arterial pressure (2e), central pulse pressure variation (2f) as well as on receiver operating characteristic analysis using central pulse pressure variation (2g) as well as peripheral pulse pressure variation (2h).

Additional Table 2a: Statistical Details mean Flux

Case Processing Summary	N	Percent
Included	38	95,00%
Excluded	2	5,00%
Total	40	100,00%

Model Summary		
Target	mFlux	
Probability Distribution	Normal	
Link Function	Identity	
Information Criterion	Akaike Corrected	396,93
	Bayesian	399,452

Fixed Effects

Source	F	df1	df2	Sig.
Corrected Model	4,22	5	14	0,015
Point of measurement	4,59	4	26	0,006
Baseline	2,74	1	6	0,149

Fixed Coefficients

Model Term	Coefficient	Std. Error	t	Sig.	95% Confidence Interval	
					Lower	Upper
Intercept	108,81	191,41	0,57	0,59	-357,72	575,34
VLS4	-87,74	29,11	-3,01	0,006	-147,56	-27,92
VLS3	-42,48	27,90	-1,52	0,140	-99,82	14,87
VLS2	8,35	29,11	0,29	0,777	-51,48	68,17
VLS1	20,96	27,90	0,751	0,459	-36,38	78,30
I/R	0b	.	.	.	.	.
Baseline	0,42	0,25	1,66	0,149	-0,20	1,04

b This coefficient is set to zero because it is redundant.

Covariance Parameters Summary

Covariance Parameters	Residual Effect	1
	Random Effects	1
Design Matrix Columns	Fixed Effects	7
	Random Effects	1a
Common Subjects		8

Common subjects are based on the subject specifications for the residual and random effects and are used to chunk the data for better performance.

a This is the number of columns per common subject.

Residual Effect

Residual Effect	Estimate	Std. Error	Z	Sig.	95% Confidence Interval	
					Lower	Upper
Variance	3112,90	862,92	3,61	0,000	1808,03	5359,51

Covariance Structure: Scaled Identity

Subject Specification: (None)

Random Effect Covariance	Estimate	Std. Error	Z	Sig.	95% Confidence Interval	
					Lower	Upper
Var(Intercept)	18131,70	10834,45	1,67	0,094	5620,96	58488,01

Covariance Structure: Variance components

Subject Specification: Animal

Estimates	Mean	Std. Error	95% Confidence Interval	
			Lower	Upper
VLS4	327,21	52,20	206,95	447,48
VLS3	372,48	51,54	252,71	492,25
VLS2	423,30	52,20	303,04	543,57
VLS1	435,92	51,54	316,15	555,69
I/R	414,95	51,54	295,18	534,72

Pairwise contrast	Contrast Estimate	Std. Error	t	df	Adj. Sig.	95% Confidence Interval	
	Lower	Upper					
VLS4-VLS3	-45,26	29,11	-1,56	26	0,132	-105,09	14,56
VLS4-VLS2	-96,09	30,35	-3,17	26	0,004	-158,45	-33,72
VLS4-VLS1	-108,70	29,11	-3,74	26	0,001	-168,52	-48,88
VLS4-I/R	-87,74	29,11	-3,01	26	0,006	-147,56	-27,92
VLS3-VLS4	45,26	29,11	1,56	26	0,132	-14,56	105,09
VLS3-VLS2	-50,82	29,11	-1,75	26	0,093	-110,65	9,00
VLS3-VLS1	-63,44	27,90	-2,27	26	0,031	-120,78	-6,10
VLS3-I/R	-42,48	27,90	-1,52	26	0,140	-99,82	14,87
VLS2-VLS4	96,09	30,35	3,17	26	0,004	33,72	158,45
VLS2-VLS3	50,82	29,11	1,75	26	0,093	-9,00	110,65
VLS2-VLS1	-12,61	29,11	-0,43	26	0,668	-72,44	47,21
VLS2-I/R	8,35	29,11	0,29	26	0,777	-51,48	68,17
VLS1-VLS4	108,70	29,11	3,74	26	0,001	48,88	168,52
VLS1-VLS3	63,44	27,90	2,27	26	0,031	6,10	120,78
VLS4-VLS2	12,61	29,11	0,43	26	0,668	-47,21	72,44
VLS1-I/R	20,96	27,90	0,75	26	0,459	-36,38	78,30
I/R-VLS4	87,74	29,11	3,01	26	0,006	27,92	147,56
I/R-VLS3	42,48	27,90	1,52	26	0,140	-14,87	99,82
I/R-VLS2	-8,35	29,11	-0,29	26	0,777	-68,17	51,48
I/R-VLS1	-20,96	27,90	-0,75	26	0,459	-78,30	36,38

Additional Table 2b: Statistical Details Cardiac Output

Case Processing Summary	N	Percent
Included	34	85,00%
Excluded	6	15,00%
Total	40	100,00%

Model Summary		
Target	CO	
Probability Distribution	Normal	
Link Function	Identity	
Information Criterion	Akaike Corrected	74,27
	Bayesian	76,456

Fixed Effects

Source	F	df1	df2	Sig.
Corrected Model	4,66	5	12	0,014
Point of measurement	5,64	4	23	0,003
Baseline	0,75	1	5	0,427

Fixed Coefficients

Model Term	Coefficient	Std. Error	t	Sig.	95% Confidence Interval	
					Lower	Upper
Intercept	3,62	2,26	1,60	0,169	-2,16	9,40
VLS4	1,16	0,34	3,46	0,002	0,47	1,85
VLS3	1,24	0,32	3,88	0,001	0,58	1,90
VLS2	1,31	0,32	4,09	0,000	0,65	1,97
VLS1	0,83	0,32	2,59	0,016	0,17	1,49
I/R	0b	.	.	.	.	.
Baseline	-0,60	0,70	-0,86	0,427	-2,39	1,19

b This coefficient is set to zero because it is redundant.

Covariance Parameters Summary		
Covariance Parameters	Residual Effect	1
	Random Effects	1
Design Matrix Columns	Fixed Effects	7
	Random Effects	1a
Common Subjects		7

Common subjects are based on the subject specifications for the residual and random effects and are used to chunk the data for better performance.

a This is the number of columns per common subject.

Residual Effect

Residual Effect	Estimate	Std. Error	Z	Sig.	95% Confidence Interval	
					Lower	Upper
Variance	0,36	0,11	3,40	0,001	0,20	0,64

Covariance Structure: Scaled Identity

Subject Specification: (None)

Random Effect Covariance	Estimate	Std. Error	Z	Sig.	95% Confidence Interval	
					Lower	Upper
Var(Intercept)	0,31	0,24	1,28	0,201	0,07	1,43

Covariance Structure: Variance components

Subject Specification: Animal

Estimates	Mean	Std. Error	95% Confidence Interval	
			Lower	Upper
VLS4	2,84	0,33	2,15	3,53
VLS3	2,92	0,31	2,26	3,59
VLS2	2,99	0,31	2,33	3,65
VLS1	2,51	0,31	1,85	3,18
I/R	1,68	0,31	1,02	2,35

Pairwise contrast	Contrast Estimate	Std. Error	t	df	Adj. Sig.	95% Confidence Interval	
	Lower	Upper					
VLS4-VLS3	-0,08	0,34	-0,24	23	0,813	-0,77	0,61
VLS4-VLS2	-0,15	0,34	-0,44	23	0,664	-0,84	0,55
VLS4-VLS1	0,33	0,34	0,99	23	0,333	-0,36	1,02
VLS4-I/R	1,16	0,34	3,46	23	0,002	0,47	1,85
VLS3-VLS4	0,08	0,34	0,24	23	0,813	-0,61	0,77
VLS3-VLS2	-0,07	0,32	-0,21	23	0,836	-0,73	0,59
VLS3-VLS1	0,41	0,32	1,29	23	0,211	-0,25	1,07
VLS3-I/R	1,24	0,32	3,88	23	0,001	0,58	1,90
VLS2-VLS4	0,15	0,34	0,44	23	0,664	-0,55	0,84
VLS2-VLS3	0,07	0,32	0,21	23	0,836	-0,59	0,73
VLS2-VLS1	0,48	0,32	1,50	23	0,148	-0,18	1,14
VLS2-I/R	1,31	0,32	4,09	23	0,000	0,65	1,97
VLS1-VLS4	-0,33	0,34	-0,99	23	0,333	-1,02	0,36
VLS1-VLS3	-0,41	0,32	-1,29	23	0,211	-1,07	0,25
VLS4-VLS2	-0,48	0,32	-1,50	23	0,148	-1,14	0,18
VLS1-I/R	0,83	0,32	2,59	23	0,016	0,17	1,49
I/R-VLS4	-1,16	0,34	-3,46	23	0,002	-1,85	-0,47
I/R-VLS3	-1,24	0,32	-3,88	23	0,001	-1,90	-0,58
I/R-VLS2	-1,31	0,32	-4,09	23	0,000	-1,97	-0,65
I/R-VLS1	-0,83	0,32	-2,59	23	0,016	-1,49	-0,17

Additional Table 2c: Statistical Details Stroke Volume

Case Processing Summary	N	Percent
Included	34	85,00%
Excluded	6	15,00%
Total	40	100,00%

Model Summary		
Target	SV	
Probability Distribution	Normal	
Link Function	Identity	
Information Criterion	Akaike Corrected	205,76
	Bayesian	207,941

Fixed Effects				
Source	F	df1	df2	Sig.
Corrected Model	8,84	5	12	0,001
Point of measurement	10,85	4	23	0,000
Baseline	1,11	1	5	0,337

Fixed Coefficients						
Model Term	Coefficient	Std. Error	t	Sig.	95% Confidence Interval	
					Lower	Upper
Intercept	30,65	10,59	2,89	0,029	4,50	56,80
VLS4	17,03	3,30	5,16	0,000	10,21	23,86
VLS3	17,20	3,15	5,47	0,000	10,70	23,71
VLS2	16,50	3,15	5,25	0,000	10,00	23,01
VLS1	10,49	3,15	3,334	0,003	3,98	16,99
I/R	0b	.	.	.	.	.
Baseline	-0,23	0,22	-1,05	0,337	-0,78	0,32

b This coefficient is set to zero because it is redundant.

Covariance Parameters Summary		
Covariance Parameters	Residual Effect	1
	Random Effects	1
Design Matrix Columns	Fixed Effects	7
	Random Effects	1a
Common Subjects		7

Common subjects are based on the subject specifications for the residual and random effects and are used to chunk the data for better performance.
a This is the number of columns per common subject.

Residual Effect						
Residual Effect	Estimate	Std. Error	Z	Sig.	95% Confidence Interval	
					Lower	Upper
Variance	34,62	10,20	3,40	0,001	19,44	61,67

Covariance Structure: Scaled Identity
Subject Specification: (None)

Random Effect Covariance	Estimate	Std. Error	Z	Sig.	95% Confidence Interval	
					Lower	Upper
Var(Intercept)	13,66	13,21	1,04	0,301	2,06	90,84

Covariance Structure: Variance components
Subject Specification: Animal

Estimates	Mean	Std. Error	95% Confidence Interval	
			Lower	Upper
VLS4	36,78	2,80	30,97	42,60
VLS3	36,95	2,63	31,47	42,44
VLS2	36,25	2,63	30,76	41,74
VLS1	30,23	2,63	24,75	35,72
I/R	19,75	2,63	14,26	25,24

Pairwise contrast	Contrast Estimate	Std. Error	t	df	Adj. Sig.	95% Confidence Interval	
	Lower	Upper					
VLS4-VLS3	-0,17	3,30	-0,05	23	0,959	-6,99	6,65
VLS4-VLS2	0,53	3,30	0,16	23	0,873	-6,29	7,35
VLS4-VLS1	6,55	3,30	1,99	23	0,059	-0,27	13,37
VLS4-I/R	17,03	3,30	5,16	23	0,000	10,21	23,86
VLS3-VLS4	0,17	3,30	0,05	23	0,959	-6,65	6,99
VLS3-VLS2	0,70	3,15	0,22	23	0,825	-5,80	7,21
VLS3-VLS1	6,72	3,15	2,14	23	0,043	0,21	13,23
VLS3-I/R	17,20	3,15	5,47	23	0,000	10,70	23,71
VLS2-VLS4	-0,53	3,30	-0,16	23	0,873	-7,35	6,29
VLS2-VLS3	-0,70	3,15	-0,22	23	0,825	-7,21	5,80
VLS2-VLS1	6,02	3,15	1,91	23	0,068	-0,49	12,52
VLS2-I/R	16,50	3,15	5,25	23	0,000	10,00	23,01
VLS1-VLS4	-6,55	3,30	-1,99	23	0,059	-13,37	0,27
VLS1-VLS3	-6,72	3,15	-2,14	23	0,043	-13,23	-0,21
VLS4-VLS2	-6,02	3,15	-1,91	23	0,068	-12,52	0,49
VLS1-I/R	10,49	3,15	3,33	23	0,003	3,98	16,99
I/R-VLS4	-17,03	3,30	-5,16	23	0,000	-23,86	-10,21
I/R-VLS3	-17,20	3,15	-5,47	23	0,000	-23,71	-10,70
I/R-VLS2	-16,50	3,15	-5,25	23	0,000	-23,01	-10,00
I/R-VLS1	-10,49	3,15	-3,33	23	0,003	-16,99	-3,98

Additional Table 2d: Statistical Details Heart Rate

Case Processing Summary	N	Percent
Included	39	97,50%
Excluded	1	2,50%
Total	40	100,00%

Model Summary

Target	HR	
Probability Distribution	Normal	
Link Function	Identity	
Information Criterion	Akaike Corrected	300,63
	Bayesian	303,22

Fixed Effects

Source	F	df1	df2	Sig.
Corrected Model	4,51	5	14	0,011
Point of measurement	5,57	4	27	0,002
Baseline	0,38	1	6	0,558

Fixed Coefficients

Model Term	Coefficient	Std. Error	t	Sig.	95% Confidence Interval	
					Lower	Upper
Intercept	122,38	41,02	2,98	0,023	22,94	221,82
VLS4	-28,05	6,61	-4,24	0,000	-41,62	-14,48
VLS3	-23,89	6,34	-3,77	0,001	-36,90	-10,88
VLS2	-18,03	6,34	-2,84	0,008	-31,04	-5,02
VLS1	-14,24	6,34	-2,25	0,033	-27,24	-1,23
I/R	0b	.	.	.	.	.
Baseline	-0,37	0,60	-0,62	0,558	-1,84	1,09

b This coefficient is set to zero because it is redundant.

Covariance Parameters Summary

Covariance Parameters	Residual Effect	1
	Random Effects	1
Design Matrix Columns	Fixed Effects	7
	Random Effects	1a
Common Subjects		7

Common subjects are based on the subject specifications for the residual and random effects and are used to chunk the data for better perform

a This is the number of columns per common subject.

Residual Effect

Residual Effect	Estimate	Std. Error	Z	Sig.	95% Confidence Interval	
					Lower	Upper
Variance	160,746	43,74	3,68	0	94,30	274,01

Covariance Structure: Scaled Identity
Subject Specification: (None)

Random Effect Covariance	Estimate	Std. Error	Z	Sig.	95% Confidence Interval	
					Lower	Upper
Var(Intercept)	438,878	272,087	1,613	0,107	130,207	1479,295

Covariance Structure: Variance components
Subject Specification: Animal

Estimates	Mean	Std. Error	95% Confidence Interval	
			Lower	Upper
VLS4	69,76	8,86	50,11	89,42
VLS3	73,93	8,67	54,49	93,36
VLS2	79,79	8,67	60,35	99,22
VLS1	83,58	8,67	64,14	103,02
I/R	97,82	8,67	78,38	117,25

Pairwise contrast	Contrast Estimate	Std. Error	t	df	Adj. Sig.	95% Confidence Interval	
	Lower	Upper					
VLS4-VLS3	-4,16	6,62	-0,63	27	0,535	-17,73	9,41
VLS4-VLS2	-10,02	6,62	-1,52	27	0,141	-23,60	3,55
VLS4-VLS1	-13,82	6,62	-2,09	27	0,046	-27,39	-0,24
VLS4-I/R	-28,05	6,62	-4,24	27	0,000	-41,62	-14,48
VLS3-VLS4	4,16	6,62	0,63	27	0,535	-9,41	17,73
VLS3-VLS2	-5,86	6,34	-0,93	27	0,363	-18,87	7,14
VLS3-VLS1	-9,65	6,34	-1,52	27	0,139	-22,66	3,35
VLS3-I/R	-23,89	6,34	-3,77	27	0,001	-36,90	-10,88
VLS2-VLS4	10,02	6,62	1,52	27	0,141	-3,55	23,60
VLS2-VLS3	5,86	6,34	0,93	27	0,363	-7,14	18,87
VLS2-VLS1	-3,79	6,34	-0,60	27	0,555	-16,80	9,22
VLS2-I/R	-18,03	6,34	-2,84	27	0,008	-31,04	-5,02
VLS1-VLS4	13,82	6,62	2,09	27	0,046	0,24	27,39
VLS1-VLS3	9,65	6,34	1,52	27	0,139	-3,35	22,66
VLS4-VLS2	3,79	6,34	0,60	27	0,555	-9,22	16,80
VLS1-I/R	-14,24	6,34	-2,25	27	0,033	-27,24	-1,23
I/R-VLS4	28,05	6,62	4,24	27	0,000	14,48	41,62
I/R-VLS3	23,89	6,34	3,77	27	0,001	10,88	36,90
I/R-VLS2	18,03	6,34	2,84	27	0,008	5,02	31,04
I/R-VLS1	14,24	6,34	2,25	27	0,033	1,23	27,24

Additional Table 2e: Statistical Details Mean Arterial Pressure

Case Processing Summary	N	Percent
Included	37	92,50%
Excluded	3	7,50%
Total	40	100,00%

Model Summary		
Target	MAP	
Probability Distribution	Normal	
Link Function	Identity	
Information Criterion	Akaike Corrected	238,46
	Bayesian	240,901

Fixed Effects

Source	F	df1	df2	Sig.
Corrected Model	8,86	5	18	0,000
Point of measurement	9,90	4	25	0,000
Baseline	5,85	1	9	0,039

Fixed Coefficients

Model Term	Coefficient	Std. Error	t	Sig.	95% Confidence Interval	
					Lower	Upper
Intercept	-3,91	14,86	-0,26	0,798	-37,41	29,59
VLS4	22,46	4,86	4,62	0,000	12,54	32,38
VLS3	19,05	4,30	4,43	0,000	10,28	27,82
VLS2	18,54	3,65	5,08	0,000	11,09	26,00
VLS1	15,78	2,77	5,706	0,000	10,09	21,47
I/R	0b	.	.	.	.	.
Baseline	0,46	0,19	2,42	0,039	0,03	0,89

b This coefficient is set to zero because it is redundant.

Covariance Parameters Summary		
Covariance Parameters	Residual Effect	2
	Random Effects	0
Design Matrix Columns	Fixed Effects	7
	Random Effects	0a
Common Subjects		8

Common subjects are based on the subject specifications for the residual and random effects and are used to chunk the data for better performance.

a This is the number of columns per common subject.

Residual Effect	Estimate	Std. Error	Z	Sig.	95% Confidence Interval	
					Lower	Upper
AR1 Diagonal	117,76	43,29	2,72	0,007	57,29	242,06
AR1 Rho	0,74	0,10	7,48	0,000	0,48	0,88

Covariance Structure: First-order autoregressive

Subject Specification: Animal

Estimates	Mean	Std. Error	95% Confidence Interval	
			Lower	Upper
VLS4	52,34	4,18	43,55	61,14
VLS3	48,93	3,96	40,52	57,34
VLS2	48,43	3,86	40,20	56,66
VLS1	45,67	3,86	37,43	53,90
I/R	29,89	3,86	21,65	38,12

Pairwise contrast	Contrast Estimate	Std. Error	t	df	Adj. Sig.	95% Confidence Interval	
	Lower	Upper					
VLS4-VLS3	3,41	3,17	1,08	26	0,292	-3,11	9,93
VLS4-VLS2	3,92	4,04	0,97	30	0,341	-4,34	12,18
VLS4-VLS1	6,68	4,53	1,47	31	0,151	-2,57	15,92
VLS4-I/R	22,46	4,86	4,62	31	0,000	12,54	32,38
VLS3-VLS4	-3,41	3,17	-1,08	26	0,292	-9,93	3,11
VLS3-VLS2	0,51	2,94	0,17	26	0,865	-5,53	6,54
VLS3-VLS1	3,27	3,78	0,86	29	0,394	-4,46	11,00
VLS3-I/R	19,05	4,30	4,43	31	0,000	10,28	27,82
VLS2-VLS4	-3,92	4,04	-0,97	30	0,341	-12,18	4,34
VLS2-VLS3	-0,51	2,94	-0,17	26	0,865	-6,54	5,53
VLS2-VLS1	2,76	2,77	1,00	26	0,327	-2,93	8,45
VLS2-I/R	18,54	3,65	5,08	29	0,000	11,09	26,00
VLS1-VLS4	-6,68	4,53	-1,47	31	0,151	-15,92	2,57
VLS1-VLS3	-3,27	3,78	-0,86	29	0,394	-11,00	4,46
VLS4-VLS2	-2,76	2,77	-1,00	26	0,327	-8,45	2,93
VLS1-I/R	15,78	2,77	5,71	26	0,000	10,09	21,47
I/R-VLS4	-22,46	4,86	-4,62	31	0,000	-32,38	-12,54
I/R-VLS3	-19,05	4,30	-4,43	31	0,000	-27,82	-10,28
I/R-VLS2	-18,54	3,65	-5,08	29	0,000	-26,00	-11,09
I/R-VLS1	-15,78	2,77	-5,71	26	0,000	-21,47	-10,09

Additional Table 2f: Statistical Details Central Pulse Pressure Variation

Case Processing Summary	N	Percent
Included	35	87,50%
Excluded	5	12,50%
Total	40	100,00%

Model Summary		
Target	PPV	
Probability Distribution	Normal	
Link Function	Identity	
Information Criterion	Akaike Corrected	221,99
	Bayesian	224,262

Fixed Effects

Source	F	df1	df2	Sig.
Corrected Model	3,31	5	8	0,064
Point of measurement	4,06	4	16	0,018
Baseline	0,27	1	4	0,630

Fixed Coefficients

Model Term	Coefficient	Std. Error	t	Sig.	95% Confidence Interval	
					Lower	Upper
Intercept	22,02	6,36	3,46	0,016	6,09	37,95
VLS4	-15,25	4,85	-3,15	0,004	-25,26	-5,25
VLS3	-16,20	4,51	-3,59	0,001	-25,44	-6,95
VLS2	-14,57	4,14	-3,52	0,002	-23,05	-6,09
VLS1	-10,25	3,28	-3,13	0,006	-17,13	-3,37
I/R	0b	.	.	.	.	.
Baseline	0,47	0,89	0,52	0,630	-2,05	2,98

b This coefficient is set to zero because it is redundant.

Covariance Parameters Summary		
Covariance Parameters	Residual Effect	2
	Random Effects	0
Design Matrix Columns	Fixed Effects	7
	Random Effects	0a
Common Subjects		8

Common subjects are based on the subject specifications for the residual and random effects and are used to chunk the data for better performance.

a This is the number of columns per common subject.

Residual Effect	Estimate	Std. Error	Z	Sig.	95% Confidence Interval	
					Lower	Upper
AR1 Diagonal	85,90	34,99	2,46	0,01	38,66	190,87
AR1 Rho	0,55	0,24	2,31	0,02	-0,05	0,86

Covariance Structure: First-order autoregressive

Subject Specification: Animal

Estimates	Mean	Std. Error	95% Confidence Interval	
			Lower	Upper
VLS4	9,59	3,70	1,68	17,49
VLS3	8,64	3,48	1,12	16,17
VLS2	10,27	3,44	2,88	17,65
VLS1	14,58	3,28	7,44	21,73
I/R	24,84	3,44	17,45	32,22

Pairwise contrast	Contrast Estimate	Std. Error	t	df	Adj. Sig.	95% Confidence Interval	
	Lower	Upper					
VLS4-VLS3	0,94	3,53	0,27	18	0,792	-6,46	8,35
VLS4-VLS2	-0,68	4,28	-0,16	27	0,875	-9,46	8,10
VLS4-VLS1	-5,00	4,56	-1,10	28	0,283	-14,35	4,35
VLS4-I/R	-15,25	4,85	-3,15	24	0,004	-25,26	-5,25
VLS3-VLS4	-0,94	3,53	-0,27	18	0,792	-8,35	6,46
VLS3-VLS2	-1,63	3,31	-0,49	17	0,629	-8,61	5,36
VLS3-VLS1	-5,94	4,05	-1,47	28	0,153	-14,23	2,35
VLS3-I/R	-16,20	4,51	-3,59	28	0,001	-25,44	-6,95
VLS2-VLS4	0,68	4,28	0,16	27	0,875	-8,10	9,46
VLS2-VLS3	1,63	3,31	0,49	17	0,629	-5,36	8,61
VLS2-VLS1	-4,32	3,28	-1,32	18	0,204	-11,20	2,57
VLS2-I/R	-14,57	4,14	-3,52	27	0,002	-23,05	-6,09
VLS1-VLS4	5,00	4,56	1,10	28	0,283	-4,35	14,35
VLS1-VLS3	5,94	4,05	1,47	28	0,153	-2,35	14,23
VLS4-VLS2	4,32	3,28	1,32	18	0,204	-2,57	11,20
VLS1-I/R	-10,25	3,28	-3,13	18	0,006	-17,13	-3,37
I/R-VLS4	15,25	4,85	3,15	24	0,004	5,25	25,26
I/R-VLS3	16,20	4,51	3,59	28	0,001	6,95	25,44
I/R-VLS2	14,57	4,14	3,52	27	0,002	6,09	23,05
I/R-VLS1	10,25	3,28	3,13	18	0,006	3,37	17,13

Additional Table 2g: Statistical Details Receiver Operating Characteristic Analysis

Central Pulse Pressure Variation

Case Processing Summary

Response mFlux	Valid N (listwise)
Positive a	11
Negative	15
Missing	6

Smaller values of the test result variable(s) indicate stronger evidence for a positive actual state.
a The positive actual state is 1,00.

Area Under the Curve

Test Result Variable(s) PPV

Area	Std. Error[a]	Asymptotic Sig.[b]	Asymptotic 95% Confidence Interval	
			Lower Bound	Upper Bound
0,879	0,074	0,001	0,734	1,000

a Under the nonparametric assumption
b Null hypothesis: true area = 0.5

Coordinates of the Curve

Test Result Variable(s)

Positive if Less Than or Equal To[a]	Sensitivity	1 - Specificity	Youden Index (Sensitivity + Specificity - 1)
3,1608	0	0	0,00
4,2902	0,091	0	0,09
4,6591	0,182	0	0,18
4,9802	0,273	0	0,27
5,0762	0,364	0	0,36
5,4465	0,455	0	0,46
6,1586	0,545	0	0,55
6,5297	0,545	0,067	0,48
6,8732	0,636	0,067	0,57
7,255	0,727	0,067	0,66
**7,6677**	**0,818**	**0,067**	**0,75**
8,5735	0,818	0,133	0,69
9,1446	0,818	0,2	0,62
9,4738	0,818	0,267	0,55
11,3893	0,818	0,333	0,49
13,0212	0,818	0,4	0,42
14,3402	0,818	0,467	0,35
15,8589	0,909	0,467	0,44
16,4136	0,909	0,533	0,38
17,5641	0,909	0,6	0,31
18,9407	0,909	0,667	0,24
19,7008	1	0,667	0,33
22,5073	1	0,733	0,27
25,2853	1	0,8	0,20
34,4014	1	0,867	0,13
44,5968	1	0,933	0,07
46,8472	1	1	0,00

a The smallest cutoff value is the minimum observed test value minus 1, and the largest cutoff value is the maximum observed test value plus 1.
All the other cutoff values are the averages of two consecutive ordered observed test values.

Additional Table 2h: Statistical Details Receiver Operating Characteristic Analysis

Peripheral Pulse Pressure Variation

Case Processing Summary

Response mFlux	Valid N (listwise)
Positive a	11
Negative	15
Missing	6

Smaller values of the test result variable(s) indicate stronger evidence for a positive actual state.

a The positive actual state is 1,00.

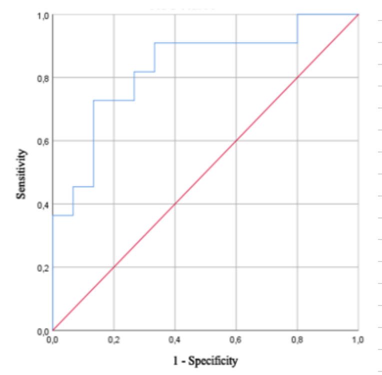

Area Under the Curve

Test Result Variable(s) PPV

Area	Std. Error[a]	Asymptotic Sig.[b]	Asymptotic 95% Confidence Interval	
			Lower Bound	Upper Bound
0,83	0,085	0,005	0,663	0,997

a Under the nonparametric assumption

b Null hypothesis: true area = 0.5

Coordinates of the Curve

Test Result Variable(s)

Positive if Less Than or Equal To[a]	Sensitivity	1 - Specificity	Youden Index (Sensitivity + Specificity - 1)
2,2992	0	0	0,00
3,9486	0,091	0	0,09
4,825	0,182	0	0,18
5,4471	0,273	0	0,27
6,0453	0,364	0	0,36
6,3902	0,364	0,067	0,30
6,702	0,455	0,067	0,39
6,9139	0,455	0,133	0,32
7,2093	0,545	0,133	0,41
7,7377	0,636	0,133	0,50
**8,0151**	**0,727**	**0,133**	**0,59**
8,4021	0,727	0,2	0,53
8,908	0,727	0,267	0,46
9,1847	0,818	0,267	0,55
9,4001	0,818	0,333	0,48
9,6612	0,909	0,333	0,58
11,3927	0,909	0,4	0,51
13,7466	0,909	0,467	0,44
18,9104	0,909	0,533	0,38
23,5468	0,909	0,6	0,31
24,3965	0,909	0,667	0,24
25,5463	0,909	0,733	0,18
28,1743	0,909	0,8	0,11
30,6064	1	0,8	0,20
38,5278	1	0,867	0,13
48,2736	1	0,933	0,07
51,4987	1	1	0,00

a The smallest cutoff value is the minimum observed test value minus 1, and the largest cutoff value is the maximum observed test value plus 1.

All the other cutoff values are the averages of two consecutive ordered observed test values.

Additional Table 3

Pairwise comparison of macro- and microcirculation with inclusion of baseline values of mean microcirculatory blood flow (mFlux), cardiac output (CO), stroke volume (SV), heart rate (HR), mean arterial pressure (MAP) and central pulse-pressure-variation (PPV). Data are presented as estimated marginal means with 95 % confidence intervals. Pairwise p-values compared to previous measurement step are given. Points of measurements are baseline conditions prior induction of ischemia/reperfusion (baseline), 6 h after ischemia/reperfusion (I/R) and volume loading steps 1–4 6 h after ischemia/reperfusion (VLS1-4).

Additional Table 3: Stepwise comparison with inclusion of baseline values for macro- and microcirculation.

mFlux (p.u.)	Mean	SD	Lower 95 % CI	Upper 95 % CI	p-value
Baseline	726,46	59,92	595,18	857,75	
I/R	413,88	59,92	282,59	545,16	<0.001
VLS 1	434,84	59,92	303,55	566,12	0.637
VLS 2	421,33	61,28	288,28	554,38	0.770
VLS 3	371,40	59,92	240,12	502,68	0.284
VLS 4	328,18	61,28	195,13	461,23	0.353

CO (l / min)	Mean	SD	Lower 95 % CI	Upper 95 % CI	p-value
Baseline	3,14	0,26	2,60	3,68	
I/R	1,66	0,28	1,09	2,24	<0.001
VLS 1	2,49	0,28	1,92	3,07	0.019
VLS 2	2,97	0,28	2,40	3,54	0.163
VLS 3	2,90	0,28	2,33	3,48	0.842
VLS 4	2,82	0,30	2,21	3,43	0.807

SV (ml)	Mean	SD	Lower 95 % CI	Upper 95 % CI	p-value
Baseline	47,52	2,54	42,36	52,69	
I/R	19,87	2,72	14,35	25,38	<0.001
VLS 1	30,35	2,72	24,83	35,87	0.008
VLS 2	36,37	2,72	30,85	41,89	0.116
VLS 3	37,07	2,72	31,55	42,59	0.851
VLS 4	36,23	2,93	30,27	42,18	0.829

HR (bpm)	Mean	SD	Lower 95 % CI	Upper 95 % CI	p-value
Baseline	66,65	7,88	50,17	83,12	
I/R	97,55	7,88	81,08	114,03	<0.001
VLS 1	83,32	7,88	66,84	99,79	0.084
VLS 2	79,53	7,88	63,05	96,00	0.638
VLS 3	73,66	7,88	57,19	90,14	0.468
VLS 4	70,68	8,21	53,63	87,72	0.722

MAP (mmHg)	Mean	SD	Lower 95 % CI	Upper 95 % CI	p-value
Baseline	75,84	5,40	64,18	87,50	
I/R	30,82	5,40	19,16	42,48	<0.001
VLS 1	46,60	5,40	34,94	58,26	<0.001
VLS 2	49,36	5,40	37,70	61,02	0.419
VLS 3	49,61	5,53	37,75	61,47	0.946
VLS 4	52,40	5,79	40,12	64,69	0.474

PPV (%)	Mean	SD	Lower 95 % CI	Upper 95 % CI	p-value
Baseline	5,90	2,69	0,43	11,37	
I/R	24,23	2,86	18,42	30,03	<0.001
VLS 1	14,52	2,69	9,06	19,99	0.011
VLS 2	9,71	2,87	3,88	15,54	0.185
VLS 3	8,24	2,88	2,40	14,08	0.689
VLS 4	8,63	3,10	2,34	14,92	0.920
